# Healthcare providers’ experiences of maternity care service delivery during the COVID-19 pandemic in the United Kingdom: a follow-up systematic review and qualitative evidence synthesis

**DOI:** 10.3389/fgwh.2024.1470674

**Published:** 2024-11-28

**Authors:** Tisha Dasgupta, Emily Bousfield, Yosha Pathak, Gillian Horgan, Lili Peterson, Hiten D. Mistry, Milly Wilson, Meg Hill, Valerie Smith, Harriet Boulding, Kayleigh S. Sheen, Aricca D. Van Citters, Eugene C. Nelson, Emma L. Duncan, Peter von Dadelszen, The RESILIENT Study Group, Laura A. Magee, Sergio A. Silverio, Laura A. Magee

**Affiliations:** ^1^ Department of Women & Children’s Health, School of Life Course & Population Sciences, King’s College London, London, United Kingdom; ^2^ School of Medicine and Population Health, Faculty of Health, University of Sheffield, Sheffield, United Kingdom; ^3^ GKT School of Medical Education, Faculty of Life Sciences & Medicine, King’s College London, London, United Kingdom; ^4^ Department of Population Health Sciences, School of Life Course & Population Sciences, King’s College London, London, United Kingdom; ^5^ The RESILIENT Study Patient & Public Involvement & Engagement Advisory Group, United Kingdom; ^6^ School of Nursing, Midwifery and Health Systems, College of Health and Agricultural Sciences, University College Dublin, Dublin, Ireland; ^7^ The Policy Institute, Faculty of Social Science & Public Policy, King’s College London, London, United Kingdom; ^8^ Department of Social Sciences, College of Health, Science and Society, University of the West of England Bristol, Bristol, United Kingdom; ^9^ The RESILIENT Study Technical Advisory Group, United Kingdom; ^10^ The Dartmouth Institute for Health Policy & Clinical Practice, Geisel School of Medicine, Dartmouth College, Hanover, NH, United States; ^11^ Department of Twin Research & Genetic Epidemiology, School of Life Course & Population Sciences, King’s College London, London, United Kingdom; ^12^ The RESILIENT Study Group, United Kingdom; ^13^ School of Psychology, Faculty of Health, Liverpool John Moores University, Liverpool, United Kingdom

**Keywords:** COVID-19, maternity services, healthcare professionals, systematic review, qualitative research

## Abstract

**Problem and background:**

During the COVID-19 pandemic, there was substantial reconfiguration of maternity care services, affecting both users and healthcare providers (HCPs), in the United Kingdom (UK) and globally.

**Aim:**

To further our understanding of the impact of maternity service reconfigurations in the UK, from the perspective of maternity HCPs.

**Methods:**

Scopus, MEDLINE, EMBASE, CINAHL, PsycINFO and the Cochrane COVID Study Register were searched for relevant studies reporting qualitative data from the UK, published in English between 01 June 2021 and 30 September 2023. Qualitative data on HCPs’ experiences of maternity care reconfiguration during the pandemic were extracted from 15 studies. Data were subjected to thematic synthesis according to key service reconfigurations.

**Results:**

Nine themes were identified: *Care-seeking and Care Experience*: Changes to existing care, Limitations placed on the partner, Mental health and lack of support networks, and Barriers to successful implementation of reconfiguration strategies; *Virtual Care*: Impact on quality of care, Increased convenience and flexibility, and Digital exclusion; and *Ethical Future of Maternity Care Services*: Optimising patient care, and Service users and staff as the driving force for change. No studies reported on the concepts of *Self-monitoring* or *COVID-19 vaccination*.

**Discussion and conclusion:**

The review findings highlight HCPs’ views of the need for greater inclusion of partners, choice of virtual or in-person care for women and birthing people; and a need for co-designed services for future policy-making.

## 1 Introduction

During the COVID-19 pandemic, maternity care was provided throughout as essential within National Health Service (NHS) provision (3). Nevertheless, substantial service reconfigurations were made.

Guidance from the Royal College of Obstetricians and Gynaecologists [RCOG] and the Royal College of Midwives [RCM] was published frequently, often updated weekly (1). Guidance aimed to: prevent transmission of the SARS-CoV-2 virus, adapt services to increased demand in acute care settings, and respond to heightened maternal vulnerability to severe COVID-19 associated with pregnancy (1). For women and birthing people, service reconfigurations included: a shift to virtual care provision for at least some antenatal care visits (2–5); fewer antenatal visits (20); alterations in some diagnostic care pathways (6, 7); exclusion of fathers, partners, and non-gestational parents from many aspects of care (3, 8–10); and restriction on choice of place of birth (11). Other changes to maternity services included: new satellite ‘Nightingale’ hospitals, reorganisation of existing hospital facilities, redeployment of maternity staff to other departments, and encouragement of newly-retired staff to return to work (2, 12, 13). Throughout the pandemic, maternity healthcare providers (HCPs) continued to work in high-risk areas, facing new challenges and rapid changes.

In their qualitative thematic synthesis of global literature, which included 17 studies published between 01 January 2020 and 13 June 2021, Flaherty et al. explored HCPs’ experiences of providing maternity care during the pandemic, identifying positive and negative impacts (14). Inconsistencies and recurrent changes in guidelines left HCPs feeling confused and unable to provide safe and effective care (14). HCP workload increased, and as the pandemic continued for longer than anticipated, acute changes became chronic. Staff burnout became evident, relating to staff shortages (15), the burden of additional tasks required to deliver new care practices, and the need for longer antenatal and postnatal appointments to address pregnant women and birthing people's questions and anxiety (14). Simultaneously, maternity HCPs reported enhanced camaraderie and bonding with colleagues, which led to a more positive working environment (14).

As the pandemic resolves, HCPs have reflected and considered the value of pandemic-related changes to maternity care (23). Thus, we updated the previous relevant systematic review (14), with literature published to September 2023, to inform future development and organisation of maternity services.

## 2 Methods

The review forms part of the RESILIENT study: Post pandemic planning for maternity care for local, regional, and national maternity systems across the four nations (NIHR134293) (16). The review was registered with PROSPERO [CRD42022355948] (17) and adheres to the PRISMA 2020 statement (18) (Supplementary Table S1).

### 2.1 Inclusion criteria

We followed the SPIDER (Sample, Phenomenon of Interest, Design, Evaluation, and Research Type) framework used in the original review (14).

Our sample included maternity HCPs directly involved in provision of maternity care during the COVID-19 pandemic. A range of professions were captured, including, but not limited to midwifery, nursing, and obstetrics. Whilst we sought studies published globally, in this review, we have restricted our sample to UK-based studies (see Search Strategy and Selection section for further detail). The phenomenon of interest was HCP experience of maternity care provision during the pandemic, including all antenatal care (except abortion), labour and childbirth, and up to six months postpartum. Care in all settings was considered. Qualitative study designs of interest included: descriptive, exploratory, and interpretive studies; ethnographic studies; observational or mixed-methods studies in which qualitative data had been extracted separately; survey designs with open-text questions when significant qualitative data had been collected and formally analysed; linguistic studies; and studies of public discourse. Only published literature was included.

Literature published between 01 June 2021 and 30 September 2023 [building on the previous review's (14) search, 01 January 2020 to 13 June 2021] was sought. The search strategy was restricted to English language.

### 2.2 Search strategy and selection

Systematic searches were undertaken of the electronic databases of Scopus, MEDLINE (Online counterpart of MEDLARS MEDical Literature Analysis and Retrieval System), EMBASE (Excerpta Medica dataBASE), CINAHL (Cumulative Index of Nursing and Allied Health Literature), PsycINFO and the Cochrane COVID Study Register. The search terms and keywords used in the Flaherty et al. review (14) were adopted (Supplementary Table S2).

EndNote Reference Manager was used to clean search result duplicates, and citations were uploaded to the Rayyan web-based systematic reviewing tool. Team members (TD, LP, GH, MW, SAS, HDM, PvD, LAM) independently screened each title and abstract, followed by full-text review. After each screening stage, disagreements were resolved through discussion with the wider team. Given the large number of studies meeting inclusion criteria, and the focus of RESILIENT on maternity care in the UK, a decision was made prior to data extraction to sub-divide the review by population of interest (women and birthing people or HCPs) and geography [UK, other high-income countries (HICs), or low- and middle-income countries]. The remaining studies have (19) or will be synthesised separately.

### 2.3 Data extraction and synthesis

Data were extracted independently by two reviewers (EB, YP) into a pre-designed Microsoft Excel data extraction sheet, and checked by wider-team members during regular discussions. Extracted information included: characteristics of studies (e.g., reference, aims, setting, and dates of data collection) and participants (e.g., number, setting), data collection method, details of analyses, and themes identified, all taken from the results sections of included papers. Then, each paper was imported into NVivo qualitative research software for coding and synthesis of Discussion sections. Of note, Results sections were not coded to avoid replicating codes/themes and rendering logic circular.

In line with the previous reviews (7, 14), methodological quality was assessed independently by two team members (EB, YP) and checked for correctness by other authors, using an adapted version of a 12-item EPPI-Centre (Evidence for Policy and Practice Information and Co-ordinating Centre) tool which captures information on the reliability and validity of study methods and reporting, for qualitative evidence synthesis (20). Data were included for synthesis, regardless of quality, to provide relevant ‘views/experiences’ data. We did, however, interrogate our final results to ensure that inclusion of low-quality papers, if any, did not compromise the integrity of resulting themes (that is, the removal of codes derived from low quality studies was not found to affect the overall set of derived themes).

Thematic Synthesis (21) was undertaken based on a set of *a priori* concepts which address RESILIENT aims: (1) Care-seeking and care experience, (2) Virtual care, (3) Self-monitoring, (4) COVID-19 vaccination, and (5) Ethical future of maternity care services. Extracted data from each study were aligned with one or more of these key concepts, then data under each concept were coded, and descriptive themes generated inductively. Data were synthesised independently by two reviewers (EB, YP), to ensure cohesion and congruity in coding, with regular discussion to resolve any conflicts and agree on derived themes.

## 3 Results

### 3.1 Search and selection

**Figure 1:**
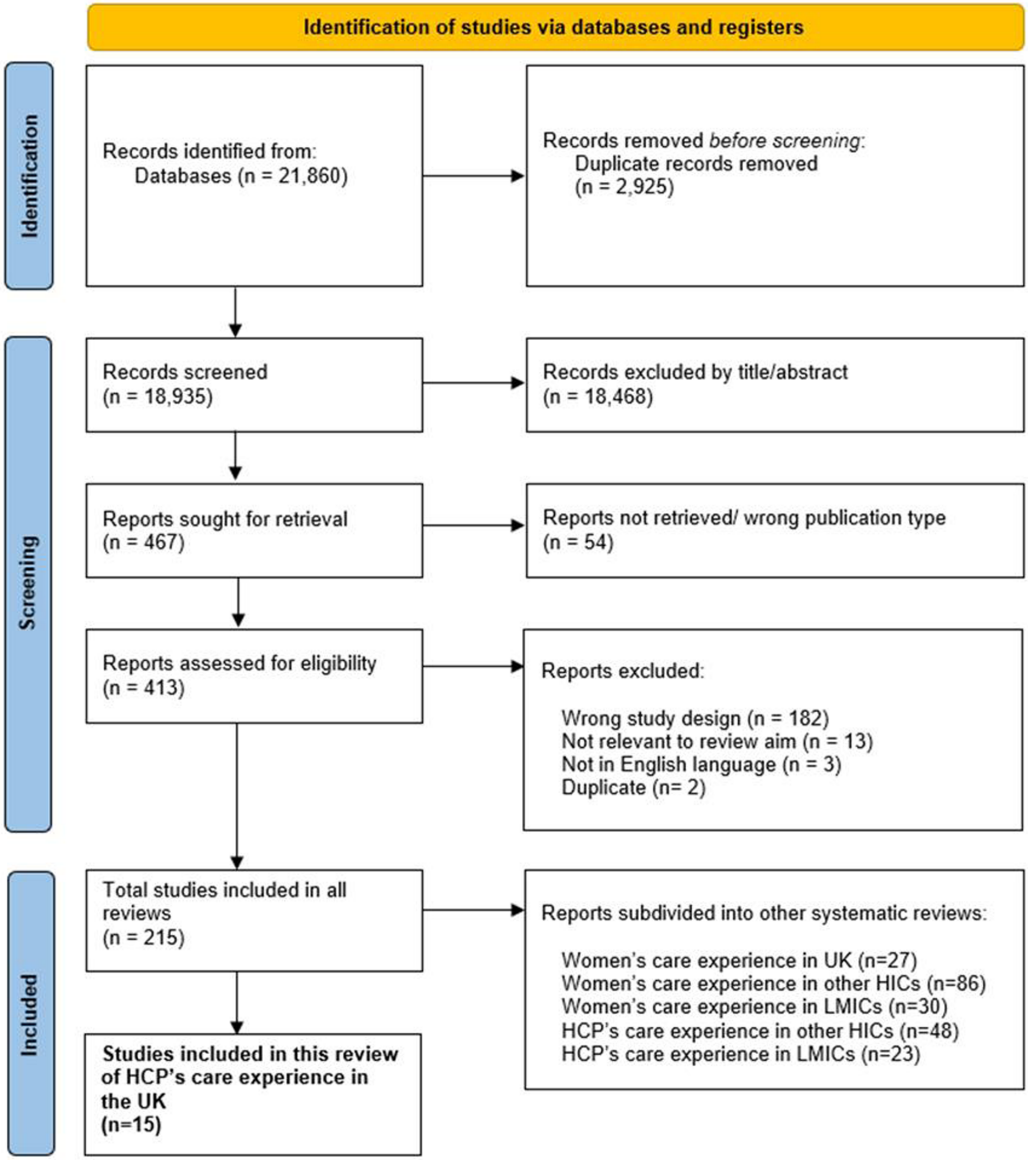
PRISMA 2020 flow diagram of study selection process.

Figure 1 illustrates the search and selection process (18). The initial literature search yielded 21,860 records. Records were removed if they were: duplicates (*n* = 2,925); ineligible at title/abstract screening (*n* = 18,468); could not be retrieved (*n* = 54); or were ineligible at full-text review (*n* = 200; see Figure 1 for reasons). Thereafter, 215 records met inclusion criteria, of which 15 studies of UK HCPs’ experiences of delivering maternity care during the pandemic are reported here (4, 5, 22–34).

### 3.2 Description of included studies

The 15 included studies were exclusively UK-based, apart from one; this compared care between the UK and the Netherlands (31), and presented data all together. One study (18) presented data from maternity and children's healthcare professionals, commenting on each set of professionals.

There were 940 participants, with additional unspecified numbers from 224 maternity units (23, 30) and eight maternity care policy organisations (30). Data collection ranged from February 2020 to November 2021, extended by 11 months beyond the original review (14). Study methodology varied: semi-structured interviews (*n* = 8) (5, 22, 24–26, 28, 29, 34), semi-structured interviews alongside analysis of policy and guidance documents (*n* = 2) (30, 31), mixed-method survey with free-text analysis (*n* = 3) (23, 27, 32), both interviews and a mixed-method survey (*n* = 1) (4), and a focus group with midwives (*n* = 1) (33). For data analysis, most studies utilised thematic analysis (*n* = 7) (22, 25–28, 32, 33); other methodologies included: content analyses (*n* = 1) (30), descriptive analysis (*n* = 2), 23,34 framework analyses (*n* = 2) (4, 31), or grounded theory analyses (*n* = 3) (5, 24, 29). For detailed characteristics of included studies and their key findings, see Supplementary Table S3.

### 3.3 Quality assessment

Study quality varied. Eight studies met all 12 quality criteria (5, 22–24, 29, 31, 33, 34); two studies met 11/12 criteria (27, 30) as they did not actively involve participants in the design and conduct of the study; one study met 10/12 criteria (4), two studies met 9/12 criteria (26, 28), one study met 8/12 criteria (25), and one study met 7/12 criteria (32). As such, all studies were deemed to be of moderate-high quality; for details, see Supplementary Table S4.

### 3.4 Synthesis and findings

Our synthesis identified nine themes within three of the five RESILIENT concepts: care-seeking and care experience, virtual care, and ethical future of maternity care services (Supplementary Table S5). No studies reported on the RESILIENT concepts of self-monitoring or COVID-19 vaccination. Passages of text from the original discussion sections are presented in Tables 1–3 to support synthesised findings.

**Table 1:** Concept 1 – care-seeking and care experience.

Themes	Quotations
Changes to existing provision of care	“This was particularly apparent during COVID-19 with conflicts between wards, services and localities. This made it particularly difficult for staff who were redeployed during the pandemic, who did not always feel included in their new ingroup but were no longer part of their old ingroup and could be left without clear lines of management support.” Billings et al. *PLoS One* 2021.
	“Some variation can probably be explained by changing national knowledge about the prevalence and impacts of COVID- 19, and by different levels of exposure to COVID- 19 infection. However, our data suggest that this was not the case where blanket policies were applied with minimal individual flexibility, or where there was unjustified variation in visiting and companionship rules, coupled with poor and inconsistent communication.” Thomson et al. *BMJ Open* 2022.
	“Relatively “simple” changes which proved possible during the COVID- 19 pandemic, such as hosting multiple clinics during the same prenatal visit, offering the choice of virtual care appointments, and allowing women more flexible access to care, created opportunities to achieve new ways of delivering high- quality care” De Backer et al. *Acta Obstetricia et Gynecologica Scandinavica* 2022.
	“Remote antenatal care can alter how women make judgments about their own care needs, complicating their ability to identify their own eligibility for health care or to make a claim for attention from the system.” Hinton et al. *Health Services Research & Policy* 2023.
Limitations placed on the partner	“In the second wave of the pandemic there continued to be signiﬁcant impacts on whether partners could attend early labour assessment (not permitted in 40% of units), be with the woman during labour (not possible in 5% of units) or visit during the womans postnatal stay (not possible in 43% of units).” Brigante et al. *Midwifery* 2022.
	“Partners may also have to leave the maternity unit shortly after the birth, and women could receive devasting news or have to make life-changing decisions without the support of their partner.” Hanley et al. *Journal of Hospital Infection* 2022.
	“Mental health care support following a miscarriage or termination or difficult birth was also largely overlooked, particularly when it came to partners. In fact, the vast majority of partners were not provided with any information or support throughout the perinatal period” Martin-Key et al. *Journal of Medical Internet Research* 2021.
Mental health and lack of support networks	“Staff often did not attend to the state of their own, and their colleagues, mental health, indicative of a lack of awareness of mental health issues in some physical healthcare settings” Billings et al. *PLoS One* 2021.
	“Medical exceptionalism promotes healthcare as an extraordinarily self-sacriﬁcing profession in which one must discount personal rights and responsibilities; in our data we saw chronic presenteeism by ethnic minority interviewees, despite risks to their own health.” Silverio et al. *eClinicalMedicine* 2022.
	Requires attention to the potential moral distress of maternity care staff (and healthcare staff in general, including ultra sonographers). These professionals are faced with the stress of having to balance these two imperatives with real people, in intensely emotional real time, repeatedly day in and day out, and at times with insufficient PPE equipment available, at a time when they too could be pregnant at risk of exposure to infection, or fearful of infecting others” Thomson et al. *BMJ Open* 2022.
	“Others who endorsed these concerns regarding the inconsistent application of care provision, explained there could be adverse psycho-social, emotional, and physical health consequences for women and for their healthcare providers” Silverio et al. *BMC Pregnancy and Childbirth* 2023.
Barriers to implementation of reconfiguration strategies	“Un(der)- preparedness and flourishing”, demonstrating fractured and fragmented services, addressed the pervasive narratives that services (and staff) were under- prepared at best, and un- prepared at worst, to cope with the magnitude of the COVID-19 health system shock” De Backer et al. *Acta Obstetricia et Gynecologica Scandinavica* 2022.
	“This was difficult due to staffing pressures, already present prior to the pandemic, which worsened due to additional tasks and a reduced workforce.” Jones et al. *BMJ Open* 2022.
	“It lacks plasticity, rendering it inﬂexible to change, and instead of facing racial and ethnic disparity head-on, it “papers over the cracks”. This is perhaps unsurprising of a system which works at “full-tilt”, 100% of the time.” Silverio et al. *eClinicalMedicine* 2022.
	“Especially problematic in situations where clinicians are having to rely more on service users when it comes to noticing and reporting symptoms, and where they do not always have ready access to complete records.” Hinton et al. *Health Services Research & Policy* 2023.
	“Most staff commented how the service was not ready to be challenged by such a significant shock, and unprepared, such as with regards to digital technology.” Silverio et al. *BMC Pregnancy Childbirth* 2023.

**Table 2:** Concept 2 – virtual care.

Themes	Quotations
Impact on quality of care	“Remote consultations may also reshape the nature and quality of the relationships between maternity service users and staff, and impact on how clinicians evaluate and make judgements about care needs. Our study suggests that continuity of care, already problematic prepandemic, may be even more challenging to achieve remotely, despite its known benefits.” Hinton et al. *Health Services Research & Policy* 2023.
	“Concerns about safety, effectiveness and person- centredness, linked to the risk that absence of in- person contact might undermine the quality of interactions and hinder safeguarding and recognition of other safety issues.” Hinton et al. *BMJ Quality & Safety* 2022.
	“PHCPs also highlighted concerns that remote assessments could not provide the same level of detail as face-to-face assessments, which may lead to misdiagnosis” Moltrecht et al. *BMC Health Services Research* 2022.
Increased convenience and flexibility	“Although participants valued the potential convenience and flexibility offered by remote care, what may appear to be efficiency gains may also involve hidden burdens leading to invisible work and compensatory labour” Hinton et al. *BMJ Quality & Safety* 2022.
	“A variable impact of virtual care on patient experience is in-line with other research, suggesting virtual care was enjoyed by some” Silverio et al. *BMC Pregnancy and Childbirth* 2023.
	“PHCPs also reported some positive aspects of the use of telemedicine including parents being easier to reach at times and an increase in the frequency of contacts with service users” Moltrecht et al. *BMC Health Services Research* 2022.
	“Although face-to-face groups were seen as ideal, online delivery offered opportunities to break down barriers such as geography and childcare, whilst appearing to retain many of the beneﬁts such as peer support and enhanced information-sharing.” Wiseman et al. *Midwifery* 2022.
	“There was also much concern about the potential for negative impacts of remote care on equality and inclusion, especially given disparities in digital access and variation in maternity outcomes linked to structural inequalities” Hinton et al. *BMJ Quality & Safety* 2022.
	“PHCPs also identified internet and mobile data charges as a significant barrier to many young parents ability to engage with telemedicine.” Moltrechet et al. *BMC Health Services Research* 2022.
Digital exclusion	“There was also much concern about the potential for negative impacts of remote care on equality and inclusion, especially given disparities in digital access and variation in maternity outcomes linked to structural inequalities” Hinton et al. *BMJ Quality & Safety* 2022.
	“PHCPs also identified internet and mobile data charges as a significant barrier to many young parents ability to engage with telemedicine.” Moltrechet et al. *BMC Health Services Research* 2022.
	“Women may not know what is expected of them in antenatal care and where socially disadvantaged women may lack knowledge or resources for digital technology, delays, and poor quality care may result.” Hinton et al. *Health Services Research & Policy* 2023.

**Table 3:** Concept 3—building an ethical future for maternity care.

Themes	Quotations
Optimising patient care	“Relatively “simple” changes which proved possible during the COVID- 19 pandemic, such as hosting multiple clinics during the same prenatal visit, offering the choice of virtual care appointments, and allowing women more flexible access to care, created opportunities to achieve new ways of delivering high- quality care” De Backer et al. *Acta Obstetricia et Gynecologica Scandinavica* 2022.
	“Optimising remote care for the future will require investment in high quality technology infrastructure, human resources and digital literacy skills and in codesigning pathways, work systems, workflows and processes to support efficiency and convenience for both service users and healthcare professionals.” Hinton et al. *BMJ Quality & Safety* 2022.
	“Policy and practice should consider whether the increased responsibilisation implied by remote antenatal care is suitable for all and ensure adequate alternative services are provided.” Hinton et al. *Health Services Research & Polic*y 2023.
	“When issuing guidance and its updates, consideration is needed of the balance required of the need for up-to-date information, with both the need for clear, consistent messaging (particularly when time is short) and the time required to implement change. Following a more reflective process should help to sustain high-quality care, and improve staff morale throughout health system shocks” Silverio et al. *BMC Pregnancy and Childbirth* 2023.
	“Our study highlighted challenges to remote consulting unique to the perinatal period. Face-to-face assessment is necessary in high-risk cases as highlighted by the recent confidential enquiry of maternal deaths in the UK during the first 3 months of the pandemic, which included four suicides and two domestic homicides” Wilson et al. *Archives of Womens Mental Health* 2021.
	“It is time to capitalise on these learnings, so that staff providing care do not feel burdened by providing care they believe to be sub-optimal, are motivated by innovation, and avoid feeling like they are in a “parrotocratic” situation whereby they are simply repeating policy handed down to them by senior Trust and Governmental sources, for whom they are expected to be an obedient mouthpiece.” Silverio et al. *BMC Pregnancy and Childbirth* 2023.
Service users and staff as the driving force for change	“Consultation and co-production with frontline staff is going to be essential in establishing systems of support which are likely to be most effective, acceptable, and sustainable.” Billings et al. *PLoS One* 2021.
	“Remote antenatal care services should be optimised for equality, inclusion and diversity and, critically, co-designed with maternity service users and representation from minoritised and marginalised groups to achieve this goal.” Hinton et al. *Health Services Research & Policy* 2023.
	“Staff wish to be engaged in care policy and planning as well as delivery, including in the process of rapid change which must be implemented at pace (i.e., re-development, re-organisation, and re-deployment)” Silverio et al. *BMC Pregnancy and Childbirth* 2023.
	“Our study emphasises that any lasting shift to remote provision will need to be highly attentive to designing care pathways so that they facilitate successful relationships between people who are pregnant and those who are caring for them” Hinton et al. *BMJ Quality & Safety* 2022
	“Staff often have valid concerns, and they must feel able to express them through existing institutional feedback mechanisms that are meaningful, timely, and most importantly, fair.” Silverio et al. *eClinicalMedicine* 2022
	“The pandemic brings into sharp focus the fundamental and underpinning ethical dilemma between social actions that ensure the greatest benefit for the population as a whole, and the individual human rights of each person within that population. Resolving this potential conflict of ethical imperatives depends on an open and informed debate about rights and consequences.” Thomson et al. *BMJ Open* 2022.

### 3.5 Concept 1: care-seeking and care experience

Fourteen studies (4, 5, 22–34) contributed data to this concept, with four themes: 1.1 Changes to existing provision of care, 1.2 Limitations placed on the partner, 1.3 Mental health and lack of support networks, and 1.4 Barriers to implementation of reconfiguration strategies (Table 1 for supportive quotations).

#### 3.5.1 Changes to existing provision of care

Studies described a reduction in midwifery-led care due to closure of community-based services and a move towards centralised obstetric-led and hospital-based care (23, 28, 31, 32). These changes were perceived to cause a reduction in mothers’ and gestational parents’ choice in birth planning, as well as emotional distress (22, 26, 31, 32). There were challenges in providing care, given rapid changes to protocols, lack of adequate guidance for staff regarding implementation (5, 23, 25), and less time allotted for discussion of care plans (23, 24, 26, 30, 34). However, one study found evidence of some positive effects: staff adapted rapidly, particularly in later lockdowns (11).

#### 3.5.2 Limitations placed on the partner

Studies showed distinct variation between hospitals in visitation rights and involvement of fathers, partners, and non-gestational parents during care appointments and birth (23, 25–27, 30, 31). Exclusion of these individuals was perceived as having negative and adverse effects on women and birthing people's healthcare experiences and emotional state. One study described how paternal mental health was often unaddressed when poor maternal or neonatal outcomes occurred (27).

#### 3.5.3 Mental health and lack of support networks

Nine contributing studies (5, 22, 24–28, 32) outlined the impact of reconfigurations on staff mental health, independent of fathers', partners', and non-gestational parents’ involvement described above. Some staff described a negative impact on morale because they could not provide the standard of care and enhanced support that their service-users deserved (22, 24–28, 32). Particular concerns were raised about a lack of guidance for staff about how they could address service-users’ perinatal mental health (27, 30, 32). Importantly, several studies described HCPs selflessly prioritising the physical and mental health of women and birthing people over their own (22, 26, 30).

#### 3.5.4 Barriers to implementation of reconfiguration strategies

Data from nine studies (5, 23, 24, 26, 28, 29, 31, 33, 34), illustrated how challenges for staff were exacerbated by the pandemic, rather than created anew (24, 26, 29, 33). The “unrealistic work pressures” (24), highlighted across studies, were attributed to increased work demands, reduced staffing, finite resources, and limited guidance on how to adjust practice and cope with difficulties (24, 26). These made it difficult for staff to adapt to new and ever-changing policies, impeding successful implementation of reconfiguration strategies.

### 3.6 Concept 2: virtual care

Nine studies (4, 5, 23, 25, 27, 28, 32–34) contributed data to this concept, describing how some maternity care changed from in-person to virtual, by telephone or video-conference. Three themes were identified: 2.1 Impact on quality of care, 2.2 Increased convenience and flexibility, and 2.3 Digital exclusion (Table 2 for supportive quotations).

#### 3.6.1 Impact on quality of care

In seven studies (4, 5, 23, 25, 27, 28, 34), concerns were raised about potential harmful consequences of virtual (vs. in-person) delivery on quality of care. HCPs felt positive and trusting patient-provider relationships were harder to establish during virtual care (23, 27, 28, 34). Patient safety was questioned with particular reference to mental health assessments; HCPs felt sensitive information might be less likely to be divulged by service-users over telephone or video calls. Additionally, concerns were expressed for children's welfare in the absence of a full assessment of home circumstances or domestic violence (4, 25, 28).

#### 3.6.2 Increased convenience and flexibility

In contrast, the shift to virtual care had some benefits. Some HCPs commented on being able to provide greater continuity of care, and more frequent contact with service-users (28).

#### 3.6.3 Digital exclusion

HCPs perceived the main barrier service-users faced accessing virtual maternity care was their limited access to the internet and/or electronic devices (including smartphones) (4, 25, 27, 28, 34), as well as limited technology skills and English-language skills (25, 27). Also, HCPs described their own difficulties with access, such as lack of compatible software resources on home devices, and unsuitable home-working environments which hindered hybrid-working (4, 5, 25, 27, 28). Others suggested the transition to virtual care required additional work and time to operationalise (4, 5).

### 3.7 Concept 3: ethical future of maternity care services

All fifteen studies (4, 5, 22–34) provided data for this concept, describing how maternity services should be built back in a fairer and ethical way, to prevent further exacerbation of health inequities. This concept was coded into two themes: 3.1 Optimising patient care, and 3.2 Service users and staff as the driving force for change (Table 3 for supportive quotations).

#### 3.7.1 Optimising patient care

Hybrid care delivery was described by HCPs as giving mothers and gestational parents the opportunity to choose face-to-face appointments should they wish (24, 25, 32), increasing their autonomy and potentially, their satisfaction with care (4). To facilitate a move to hybrid delivery, HCPs emphasised the need for adequate technology and for digital inequities to be addressed, to prevent exclusion of certain groups (service-users and staff) (27, 28, 34). Nevertheless, HCPs emphasised the need to retain in-person care for high-risk and vulnerable women and birthing people, such as those with complex medical, physical, or social needs (5, 32).

#### 3.7.2 Service-users and staff as the driving force for change

Finally, to build an ethical future of maternity care services, HCPs reported it was crucial to involve staff and service-users in policy-making, particularly through collaboration which considered local context and its challenges and opportunities. Input from those with lived experience of maternity care was seen as vital to ensure service delivery kept their needs and values at the forefront (4, 27, 31, 34). Involvement and consideration of at-risk and vulnerable populations was emphasised, particularly in times of crisis (5, 31).

## 4 Discussion

### 4.1 Main findings

This systematic review of 15 qualitative studies (4, 5, 22–34) of HCPs’ experiences of providing maternity care during the COVID-19 pandemic in the UK, builds on a previous qualitative evidence synthesis (14).

Key findings included reduction in provision of community midwifery services leading to perceived loss of autonomy for women and birthing people, challenges with providing good quality care, and inadequate guidance and support for staff regarding protocol changes and safety measures. Altered care-provision and limitations placed on the role of fathers, partners, and non-gestational parents during appointments and birth were perceived by HCPs to be detrimental for women's emotional and physical wellbeing. Staff reported loss of morale, unrealistic work pressures, and reduced staffing—making it difficult to successfully implement reconfiguration strategies.

Studies evaluating experiences of virtual care highlighted HCPs’ concerns about care quality, compared to in-person care, especially for high-risk and vulnerable groups. Participants felt that comprehensive mental health and wellbeing assessments cannot be completed virtually, potentially jeopardising women and birthing peoples’ safety, and leaving staff feeling they were unable to fulfil their duty of care. Access to digital devices and reliable internet connectivity were highlighted as problematic. However, HCPs expressed that virtual care increased convenience and flexibility, and some HCPs found it easier to provide continuity of care and more frequent contact with women and birthing parents.

HCPs perceive an ethical future for maternity care services in the UK to include: personalisation of care to suit individuals’ needs, the offer of in-person care when necessary, and the offer of hybrid care for others who prefer to avoid coming to hospital. The synthesis emphasised the importance of a co-designed and collaborative approach to designing future maternity care, by including both service-users and HCPs in the decision-making process.

### 4.2 Interpretation

To our knowledge, this is the only UK-focused systematic review of HCPs’ qualitative experiences of delivering maternity care during all three COVID-19 pandemic lockdowns.

We add 15 UK publications (4, 5, 22–34) to the single UK study included in the previous qualitative thematic synthesis by Flaherty et al. (14), comprehensively enhancing our understanding of the impact of the COVID-19 pandemic on UK HCPs’ experiences of providing care during an unprecedented health system shock. Adhering to the aims of RESILIENT, data were synthesised according to our five pre-defined concepts; however, our findings resonate with those of HCPs internationally. The six themes identified in Flaherty et al.'s review drawing on the global literature align primarily with our core concepts of Care-seeking and care experience: altered maternity care, altered care structures and provision, capacity to provide care, professional and personal impact, professional impact, and personal burden (14). We expand, by adding themes related to virtual care and, importantly, HCPs’ views on an ethical future for UK maternity care services (7). However, no data were found to align with the RESILIENT concepts of Self-monitoring or Vaccination.

Our findings align with those of service-users and specific groups of HCPs studied by other researchers. An online survey of parents in Northern England found a reduction in women and birthing people's choices and autonomy over their care (particularly with respect to birth-planning), which jeopardised their overall satisfaction and wellbeing (35). Others reported how it was difficult for HCPs to work in ways which incorporated infection control measures, whilst meeting the needs of women and birthing people, particularly given restricted personal engagement and the ability to provide supportive touch (36, 37). The negative consequences of restrictions placed on fathers', partners', and non-gestational parents’ involvement have been echoed in several other works (6, 38, 39), including potentially reducing these individuals’ ability to bond with their baby, and to offer support to the mother or gestational parent (40)

A key finding of our review was the challenge faced by staff in fulfilling their duty of care, in the face of staff shortages and limited resources. This issue has been recognised and debated by the UK Government (41). Staff surveys in the global setting have attributed staff shortages to heightened stress levels and burn-out (42). Several studies document an increase in depression, anxiety, and stress among HCPs during the pandemic, along with post-traumatic stress symptoms (43, 44).

Our findings that staff had concerns about developing trusting and meaningful relationships with women and birthing people through telephone or video consultation was echoed by service-users, who felt virtual antenatal consultations provided impersonal care and had a negative impact on how much information women and birthing people chose to disclose to their HCP (45). Workforce surveys and those from the UK's communications regulator have associated digital poverty during the COVID-19 pandemic with disabilities and lower socioeconomic background and housing tenure (46). An extensive narrative review of telemedicine in the United States of America emphasised the need for equitable access to digital technology, as well as its potential (47). With a global shift to virtual care delivery in a post-pandemic world, it is crucial to carefully consider the ramifications of using digital technology for groups that are already marginalised and prone to digital exclusion (34). It is crucial to understand the multilayered aspects involved in the adoption and implementation of this technology, from the perspective of all stakeholders, and it should not replace traditional face-to-face care, but rather complement it (51, 52). Finally, the strong desire of women and birthing people to have a model of maternity care that supports women-led decision making (48) speaks to the collaborative working and co-design expressed by HCPs in the literature we reviewed.

Although the roll-out of the COVID-19 vaccination program began in the UK in December 2020, and management of routine self-monitoring of symptoms for pregnancy complications (such as gestational diabetes and hypertension) were major maternity service reconfigurations during the pandemic, the paucity of literature reporting HCPs’ views on these concepts may indicate that staff did not perceive them to have had a major impact on their day-to-day lives, or that this was not a research priority in studies with HCPs. Other research with service users has shown self-monitoring of symptoms during pregnancy to be associated with implementation and resourcing issues (49). Within the RESILIENT programme of work, we have found in interviews with HCPs, policymakers, women, and partners that vaccination, particularly mandatory vaccination programs for staff was a contentious issue (50). We would endorse future research to confirm these findings within other contexts.

### 4.3 Strengths and limitations

This review benefits from robust data extraction and synthesis, with all studies screened for inclusion independently by at least two authors; while data extraction, quality-assessment, and synthesis were conducted independently by two authors. We evaluated HCPs’ experiences of care provision over all three pandemic lockdowns in the UK. Importantly, we gathered information on how HCPs believed maternity care can be improved in the future, and their emphasis that these should be informed by their own experiences. Given the sheer volume of literature published in the last three years about the impact of the pandemic on experiences of maternity care, and the focus of RESILIENT on the pandemic in the UK, we limited the scope of this review to UK studies only. Nevertheless, Flaherty et al. (14) reported similar views of HCPs in their review of global literature; with no thematic differences between HICs and LMICs, and we plan to complete the additional RESILIENT study systematic reviews in describing longer-term experiences internationally, imminently. Whilst we can take these findings from the UK as a critical case (53) from which we can extrapolate to other settings we realise they may not be wholly generalisable to HCPs’ experiences in other parts of the world, particularly where the system is not modelled on being ‘free-at-point-of-use’. Future publications of the RESILIENT study as well as other research should focus on comparing experiential literature between different healthcare settings. Finally, although our search strategy for the population of interest included a range of professional roles within maternity care, it may not represent the whole maternity workforce in the UK, particularly as specific social determinants (such as gender, ethnicity, or geographic location of individual Trusts) were not considered. This work would be complemented by further local and context-specific research.

## 5 Conclusion

Based on our synthesis of HCPs’ experiences of providing maternity care during COVID-19 in the UK, we make the following practical recommendations:

1. Maternity services should be optimised by providing more choice in care delivery. Pandemic preparedness plans for maternity care should prevent extensive centralisation of maternity care services and removal of services such as home births, along with ensuring that harsh restrictions are not place on birth partners.
2. Future maternity services should be co-designed with staff and service-users, to reflect their collective experiences and understanding of the context in which they provide and receive care, respectively. Taking into account staff experiences in designing services has the potential to improve workplace wellbeing and maternity staff retention, thereby positively affecting women's maternity care experience.

## Patient and public involvement and engagement

This systematic review was periodically reviewed by the Patient and Public Involvement and Engagement (PPIE) group of the wider RESILIENT Study which comprises 15 participants. The group was involved throughout, from conception of the project and research questions, through to checking findings for relevance. Meetings were held three times per year, were well-attended (with at least 8/15 members present at each), and had good representation of birthing and non-birthing parents, healthcare workers, and community support, from multiple ethnic backgrounds, birth histories, and living in different parts of the UK. Each meeting allowed for in-depth discussion and reflection of the work by the wider PPIE team, with suggested changes incorporated. Additionally, one member of the PPIE team was part of the smaller authorship team of this paper and reviewed and edited this manuscript in detail.

## The RESILIENT Study Group consists of:

*Chief Investigator:* Prof. Laura A. Magee (King's College London); and *Co-Investigators:* Prof. Debra E. Bick (The University of Warwick), Dr. Harriet Boulding (King's College London), Dr. Kathryn Dalrymple (King's College London), Ms. Tisha Dasgupta (King's College London), Prof. Emma L. Duncan (King's College London), Dr. Abigail Easter (King's College London), Prof. Julia Fox-Rushby (King's College London), Miss. Gillian Horgan (King's College London), Prof. Asma Khalil (St. George's University Hospitals NHS Foundation Trust & Liverpool Women's NHS Foundation Trust), Ms. Alice McGreevy (King's College London), Dr. Hiten D. Mistry (King's College London), Prof. Eugene C. Nelson (Dartmouth College), Prof. Lucilla Poston (King's College London), Mr. Paul Seed (King's College London), Sergio A. Silverio (King's College London & University of Liverpool), Dr. Marina Soley-Bori (King's College London), Dr. Florence Tydeman (King's College London), Ms. Aricca D. Van Citters (Dartmouth College), Dr. Sara L. White (King's College London), Prof. Ingrid Wolfe (King's College London), Prof. Yanzhong Wang (King's College London), & Prof. Peter von Dadelszen (King's College London).
