# Supplementary material for: Healthcare providers’ experiences of maternity care service delivery during the COVID-19 pandemic in the United Kingdom: a follow-up systematic review and qualitative evidence synthesis
Source: Front Glob Womens Health. 2024 Nov 28;5:1470674. doi: 10.3389/fgwh.2024.1470674 (PMC11634857; doi:10.3389/fgwh.2024.1470674)
Supplement: Supplementary file 2 [file Table2.docx]

**Table S2:** Search strategy

| **Concept** | **Search terms** |
| --- | --- |
| Sample | mother OR woman OR women OR midwives OR midwife* OR nurse* OR clinician OR physician OR doctor OR obstetric* OR professional |
|  | **AND** |
| Phenomenon of Interest | (maternity ADJ care) OR healthcare OR ‘health- care’ OR matern* OR birth* OR childbirth OR pre- nan* OR labour OR labor OR antenatal OR antepar- tum OR postnatal OR postpartum OR post-partum OR puerperium AND coronavirus* OR corona virus* OR COVID-19 OR COVID OR covid OR Covid2019 OR SARS-CoV* OR SARSCov* OR new CoV* OR novel CoV* |
|  | **AND** |
| Study Design and Evaluation | experiences OR experience OR view* OR perceptions OR perception OR voices OR nar- ratives OR qualitative OR (mixed ADJ method) OR ‘grounded theory’ OR phenomenology OR ‘action research’ |
|  | **LIMITED TO** |
| Research type | Qualitative, 01 June 2021 to 13 October 2022; updated September 2023 |
