# Supplementary material for: Healthcare providers’ experiences of maternity care service delivery during the COVID-19 pandemic in the United Kingdom: a follow-up systematic review and qualitative evidence synthesis
Source: Front Glob Womens Health. 2024 Nov 28;5:1470674. doi: 10.3389/fgwh.2024.1470674 (PMC11634857; doi:10.3389/fgwh.2024.1470674)
Supplement: Supplementary file 3 [file Table3.docx]

**Table S3:** Details of included studies

| **Study author (year)** | **Study aim** | **Description of HCP participants** | **Dates study conducted** | **Data collection method** | **Data analysis method** | **Themes identified** |
| --- | --- | --- | --- | --- | --- | --- |
| Billings et al (2021) | "To qualitatively explore UK frontline health and social care workers’ own experiences and views of psychosocial support during the  pandemic." | 25 health and social staff who  had been working directly to treat patients affected by COVID-19 in any UK based health or  social care organisation | 01 Jun – 23 Jul 2020 | Remote semi-structured interviews | Reflexive thematic analysis | Self: Just getting on with it.  Family and friends: competing demands, separation and sacrifice, not in our boat.  Colleagues, peers and teams: in the same boat, tensions and transitions, burden of responsibility.  Organisational support: practical needs, information, communication and consultation  Media and the wider public: recognition and awareness, a double-edged sword, unhelpful heroic narratives.  Psychological support services: awareness, accessibility, not for me - reluctance and stigma, value of expertise |
| Brigante et al (2022) | "To describe changes to midwifery-led service provision in the UK and the Channel  Islands during the COVID-19 pandemic." | UKMidSS reporters in 102 participating FMUs and 97 AMUs, and 74 RCM Heads of Midwifery Network, including all senior service managers | Apr 2020 – Mar 2021 | Three online national surveys | Coded into themes (no formal methodology mentioned) | Midwives' safety and wellbeing, demand for homebirth, staff shortages |
| De Backer et al (2022) ‡ | “To explore the experiences of maternity services staff who provided maternity care during the SARS-CoV-2 pandemic to inform future improvements in care” | 29 maternity HCPs from Guy's and St Thomas' NHS Foundation Trust, South London | Aug – Nov 2020 | Semi-structured video interviews | Grounded Theory Analysis | “Endemic precarity: A health system under pressure”;  “A topdown approach to managing the health system shock”;  “From un(der)-prepared  to future flourishing” |
| Hanley et al (2022) | “To explore the successes and barriers to the implementation of PHE infection prevention and control guidance in English maternity units during the COVID-19 pandemic” | 16 obstetricians, midwives and neonatologists who worked in a maternity unit in England, UK | Apr-Jul 2021 | Semi-structured video interviews | Thematic analysis | Success to implementation: existing infrastructure, training satisfaction, and organisational culture.  Barriers to implementation: application of guidance, infrastructure and resources, and variances in testing arrangements |
| Hinton et al (2022) | "To characterise what  quality would look like for remote antenatal care  from the perspectives of those who use, provide and  organise it" | 106 pregnant women and 105 maternity HCPs and managers/stakeholders | Sept – Dec 2020 | Free-text online/over phone survey and remote semi-structured interviews | Framework Analysis | Effectiveness, safety, accessibility, equity and inclusion, person-centredness, and choice and continuity |
| Hinton et al., (2023) | “To contribute to filling this gap through exploring the experiences and perspectives of pregnant women, antenatal health care professionals, and system leaders using the construct of candidacy” | 34 health care professionals, 14 managers and system level stakeholders | Sept- Dec 2020 | In-depth interviews via telephone/video call | Constant comparative method using theoretical framework of Candidacy | Women's identification of candidacy for themselves and their baby, Navigation, Permeability of services, Appearing at services, Adjudications, Operating conditions and the local production of candidacy |
| Jones et al (2022) | “To explore midwives’ and maternity support workers’ perceptions of the impact of the COVID-19 pandemic on maternity services and understand factors influencing respectful maternity care” | 9 midwives and 2 maternity support workers who worked during the first year of the COVID-19 pandemic | Mar – May 2021 | Online semi-structured interviews | Reflexive Thematic Analysis | Communication of care, clinical care, and support for families |
| Martin-Key et al (2021) | "To evaluate the current state of perinatal mental health care  provision in the United Kingdom, and users’ (women and partners) and midwives’ interest in using a digital mental health assessment throughout the perinatal period." | 90 midwives | Apr – Aug 2020 | Online surveys | Thematic analysis mapped to the COM-B framework | Physical capability, psychological capability, social opportunity, physical opportunity, automatic motivation, reflective motivation |
| Moltrecht et al (2022) | “To investigate: 1) PHCPs’ perceptions of the challenges in providing care to young parents during the COVID-19 pandemic. 2) How PHCPs navigated the increased challenges in providing care during the COVID-19 pandemic” | 17 PHCPs working with young parents | Nov 2020- May 2021 | Semi-structured online/telephone interviews | Reflexive thematic analysis | PHCPs’ perceptions of how young parents’ needs were amplified during the pandemic: Working with the  challenges faced by young parents; and Specific pandemic-related challenges for young parents. Perceptions of the impact of COVID-19 on delivery of perinatal care: Delivery of  perinatal care using virtual and remote  methods; and Consequences of changes to perinatal care |
| Silverio et al (2022) ‡ | “To understand the intersection between race and ethnicity, and healthcare provision amongst maternity and children’s HCPs, during the SARS-CoV-2 pandemic” | 29 maternity and 24 children's HCPs from an NHS Trust in ethnically-diverse South London | Aug 2020 – Jul 2021 | Semi-structured interviews via video-conferencing | Ground Theory Analysis | “A System Set in Plaster”;  “The Marginalised Majority”;  “Self-Discharging Responsibility for Change-Making”;  “Slow Progress, Not No Progress” |
| Silverio et al., (2023) ‡ | “To inform ongoing discussions regarding development of healthcare policy and guidelines aiming to build back a better maternity care service for future pandemic waves, post-pandemic recovery, and future health system shocks” | 29 healthcare providers who had been involved in maternity services reconfiguration, either planning and/or provision | Aug-Nov 2021 | Semi-structured interviews via video-conferencing | Ground Theory Analysis | Reflective decision making- unique opportunities of service improvement, Pragmatic decision making- disruption care, Reactive decision making- devaluation of care |
| Thomson et al (2022) | “To explore stakeholders’ and national organisational perspectives on companionship for women/ birthing people using antenatal and intrapartum care in England during COVID-19” | 26 national governmental, professional and service-user organisation leads, 25 maternity trusts, and 8 key governmental, professional  and service-user organisations that informed national  maternity care guidance and policy | Feb – Dec 2020 | Semi-structured via videoconferencing interviews (n=26), public-facing outputs (n=25), data extraction from documents (n=78) | Descriptive content analysis | “Postcode lottery of care”;  “Confusion and stress around ‘rules’”;  “Unintended consequences”;  “Need for flexibility”;  “‘Acceptable’ time for support”;  “Loss of human rights for gain in infection control” |
| van den Berg et al (2022) | “To compare the UK and Dutch COVID-19 maternity and neonatal care responses in three key domains: choice of birthplace, companionship, and families in vulnerable situations” | National and regional level maternity care stakeholders from the UK (n=26) and the Netherlands (n=13) | Feb – Dec 2020 | Documentary analysis of national organisation policy and guidance on  COVID-19 (n=246), and semi-structured interviews with national and regional level stakeholders | Modified "Framework Method" with combined inductive and deductive approach | Choice of birthplace, companionship, women and families in vulnerable situations, focus on infection control, facilitators and barriers for personalised care, and learning how to work together during a time of crisis |
| Wilson et al (2021) | "To explore staff perceptions of the impact of the COVID-19 pandemic on mental health service delivery  and outcomes for women who were pregnant or in the first year after birth (‘perinatal’ women)" | 323 mental health care providers in the UK working with women in the perinatal period | 22 Apr – 12 May 2020 | Online mixed-methods questionnaire | Thematic analysis | Separation of women and  babies due to suspected COVID-19 infection; PPE e.g.  wearing face mask; Reduced provision from other services e.g. health visiting and social services; Less frequent and fewer face-to-face contacts; Difficulty assessing and providing support with mother-infant interaction; Difficulty actioning safeguarding  concerns; Reduced postnatal support eg. Breast feeding; Less opportunity to detect early symptoms and signs of deterioration |
| Wiseman et al (2022) | “To identify the challenges and opportunities for rolling out a bespoke model of group antenatal care called PCs within the NHS: what kind of support and training is needed and what adaptations are appropriate, including during a pandemic when face-to-face interaction is limited” | 7 midwives who facilitated PCs | Oct 2020 | Online focus group | Thematic synthesis* using ecological model of human development | “Implementing innovation” “Philosophy of care”; and “Resource management” |

‡ Participants of the De Backer et al (2022), and Silverio et al (2021; 2022) studies report on the same 29 maternity HCPs..

* We recognise that Thematic Synthesis is not actually an analytical methodology to be used on primary data, but rather a method of synthesising qualitative articles as part of the systematic review process; however, we have recorded this as the original authors have suggested they have undertaken the work.

**Abbreviations:**

AMU: Alongside Midwifery Unit

COM-B: Capability, Opportunity, and Motivation Behaviour

COVID-19: Coronavirus disease 2019 caused by the SARS-CoV-2 virus infection

FMU: Freestanding Midwifery Unit

HCP: Health Care Provider

NHS: National Health Service

PC: Pregnancy Circles

PHCP: Perinatal Health Care Professional

PHE: Public Health England

PPE: Personal Protective Equipment

RCM: Royal College of Midwives

UK: United Kingdom

UKMidSS: United Kingdom Midwifery Study System
