# Supplementary material for: Healthcare providers’ experiences of maternity care service delivery during the COVID-19 pandemic in the United Kingdom: a follow-up systematic review and qualitative evidence synthesis
Source: Front Glob Womens Health. 2024 Nov 28;5:1470674. doi: 10.3389/fgwh.2024.1470674 (PMC11634857; doi:10.3389/fgwh.2024.1470674)
Supplement: Supplementary file 4 [file Table4.docx]

**Table S4:** Quality appraisal of included studies using an adapted version of the 12-criteria tool from Evidence for Policy and Practice Information and Co-ordinating Centre (15) *

| **Paper** | **Criteria** | |  |  |  |  |  |  |  |  |  |  |
| --- | --- | --- | --- | --- | --- | --- | --- | --- | --- | --- | --- | --- |
|  | Aims and objectives clearly reported | Adequately described context of research | Adequately described sample and sampling methods | Adequately described data collection methods | Adequately described data analysis methods | Reliability of data collection tools | Validity of data collection tools | Reliability of data analysis | Validity of data analysis | Used appropriate data collection methods to allow expression of views | Used appropriate methods for ensuring analysis was grounded in the views | Actively involved participants in study design and conduct |
| Billings et al (2021) | 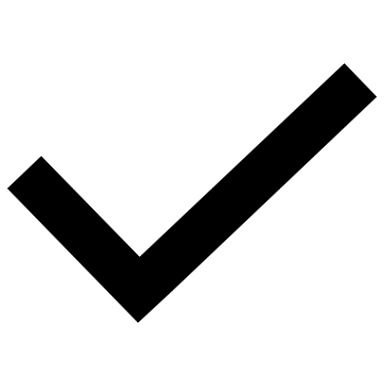 | 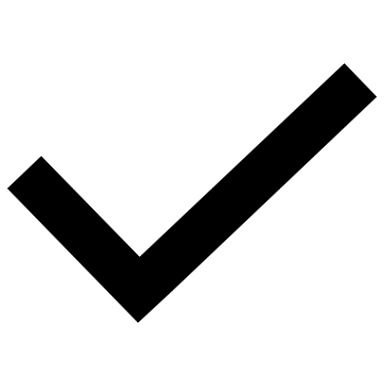 | 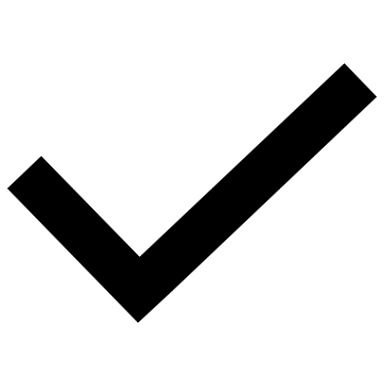 | 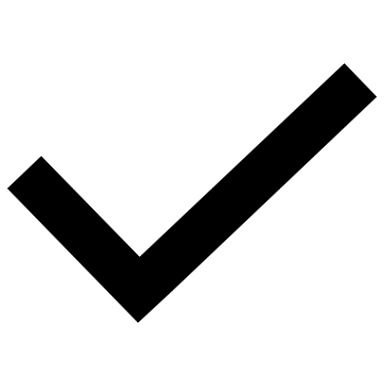 | 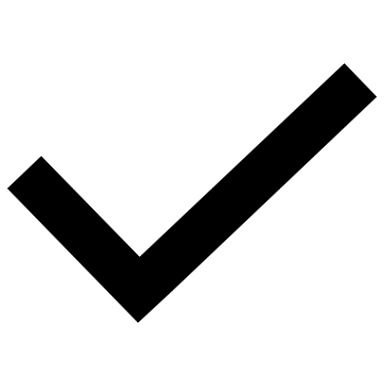 | – | 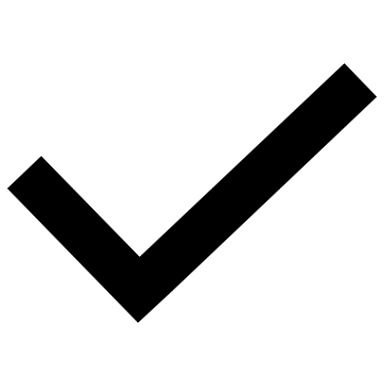 | – | 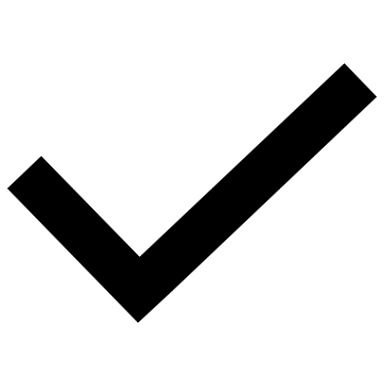 | 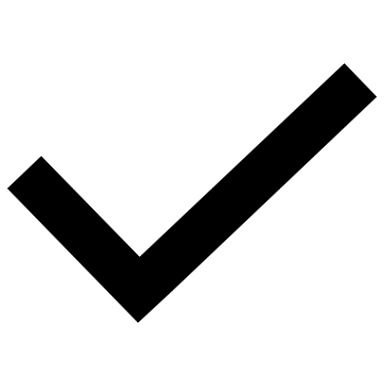 | 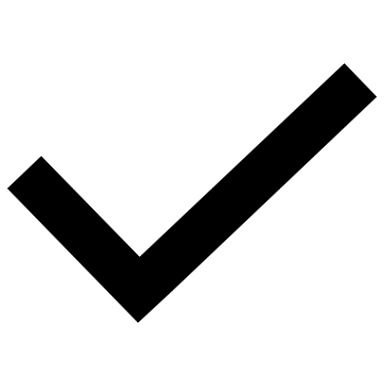 | – |
| Brigante et al (2022) | 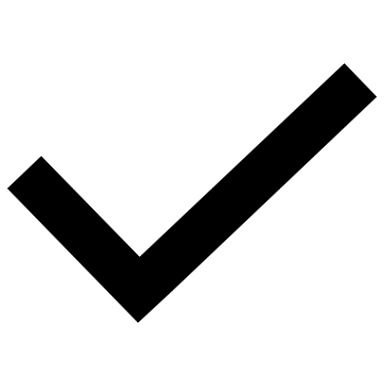 | 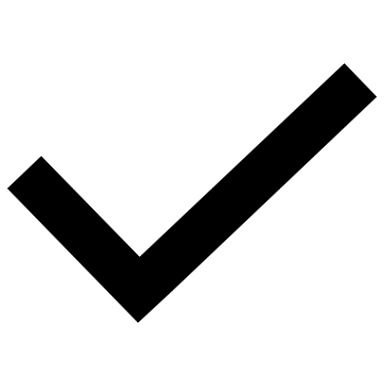 | 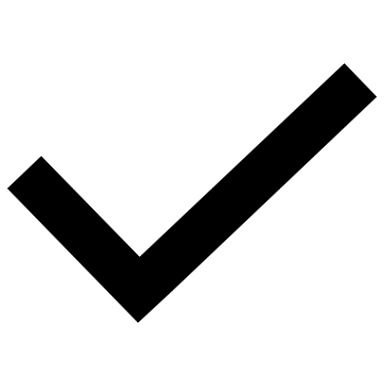 | – | 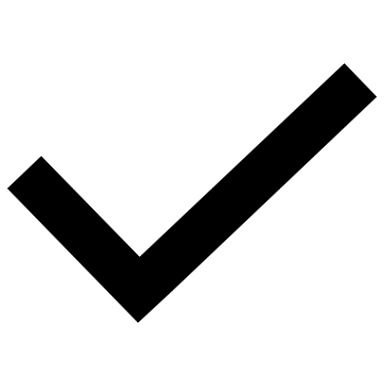 | – | 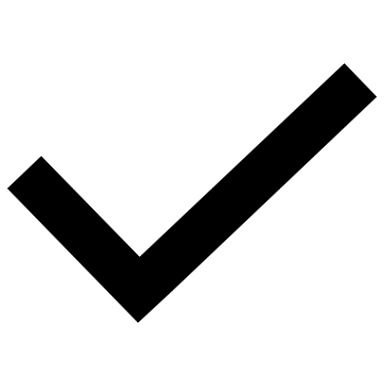 | – | 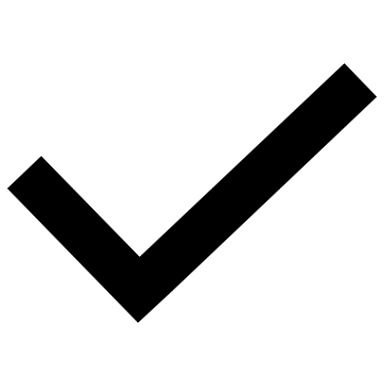 | – | 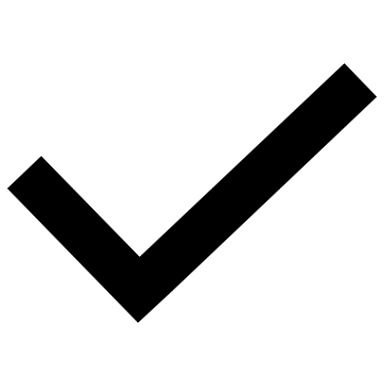 | – |
| De Backer et al (2022) | 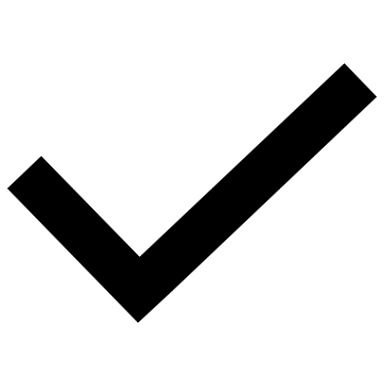 | 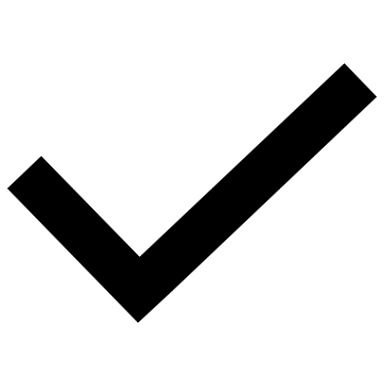 | 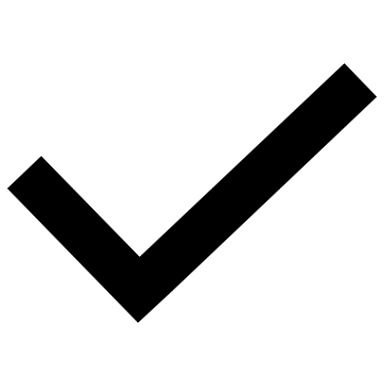 | 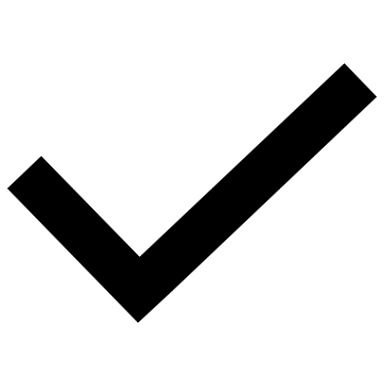 | 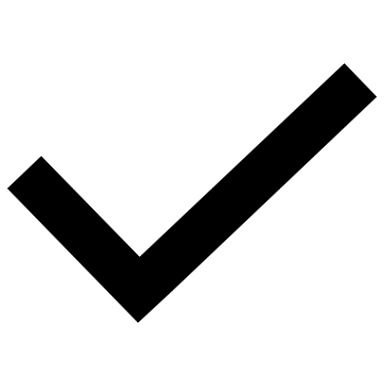 | 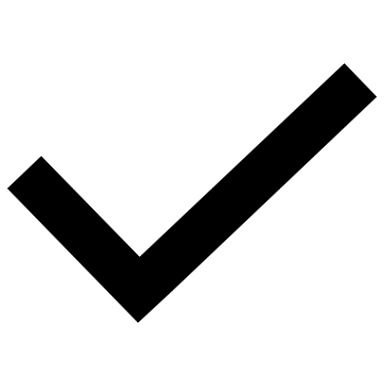 | 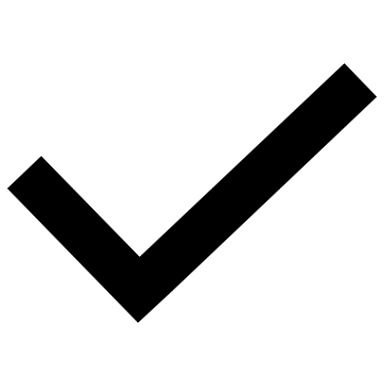 | 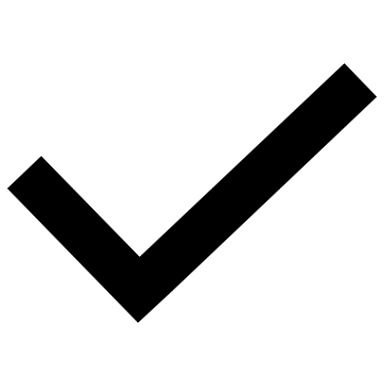 | 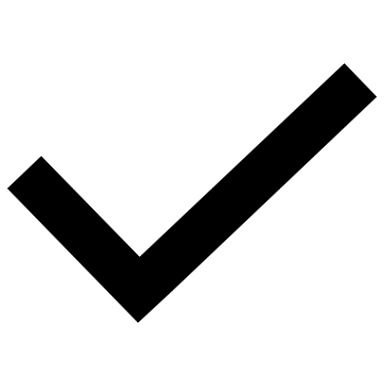 | 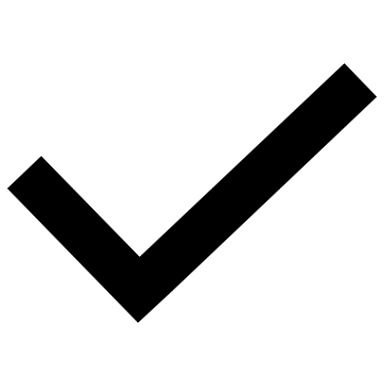 | 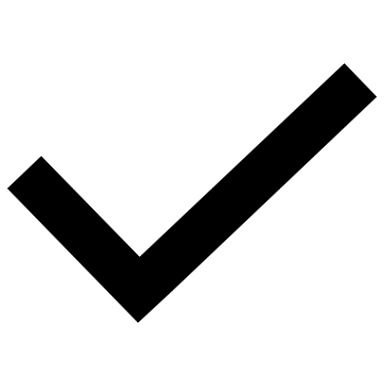 | 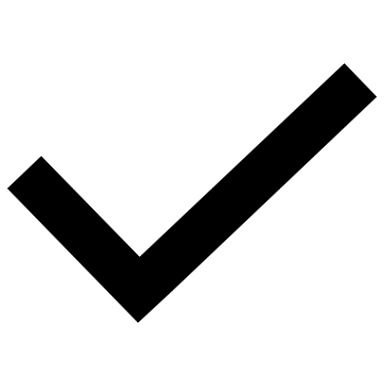 |
| Hanley et al (2022) | 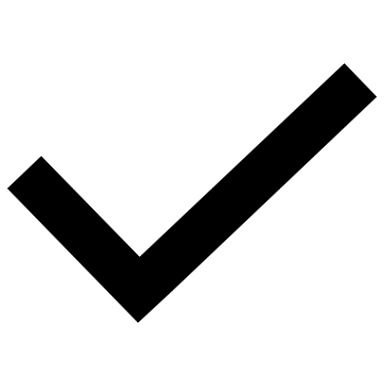 | 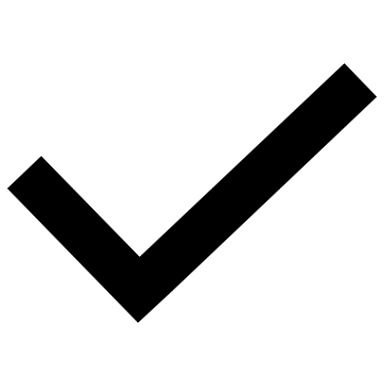 | 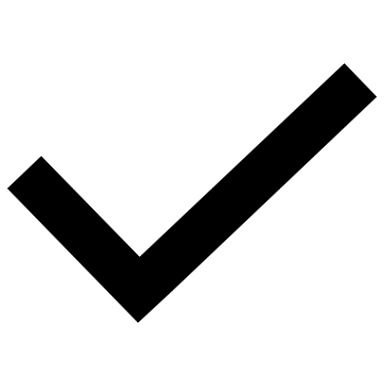 | 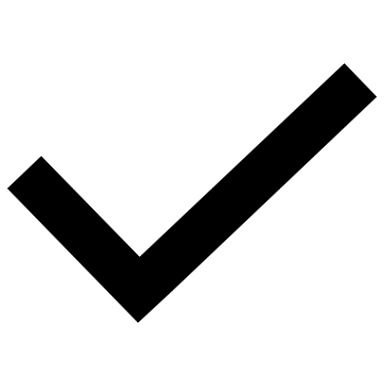 | – | 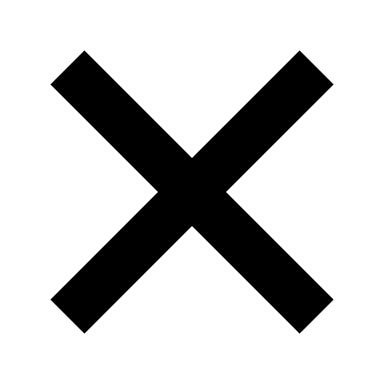 | 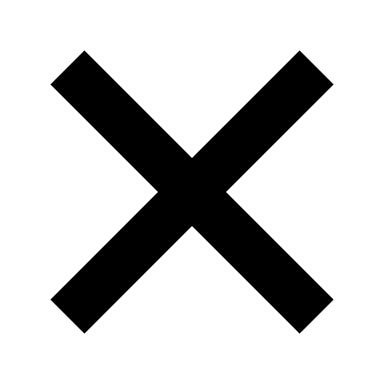 | 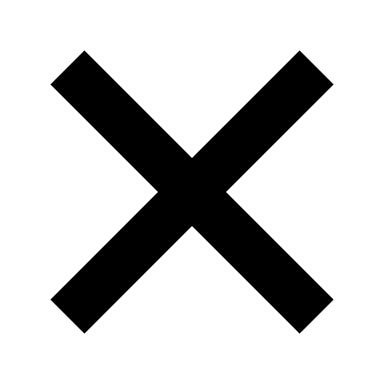 | 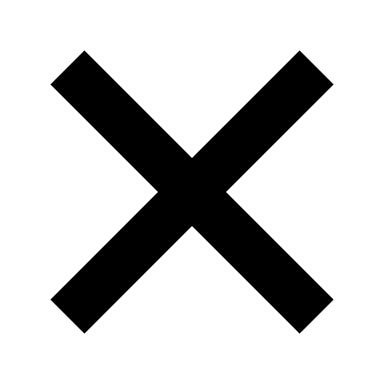 | 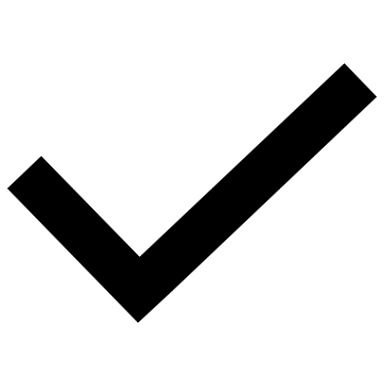 | 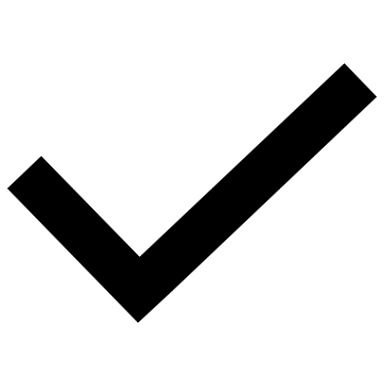 | – |
| Hinton et al (2022) | 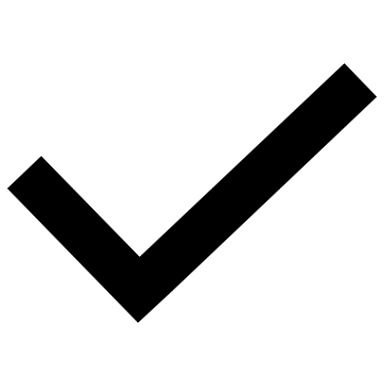 | 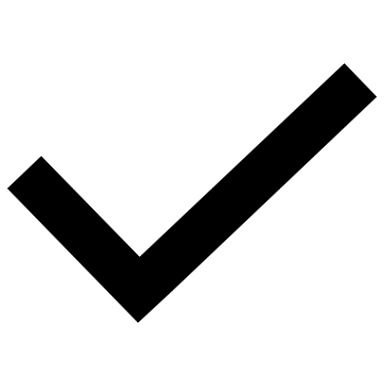 | 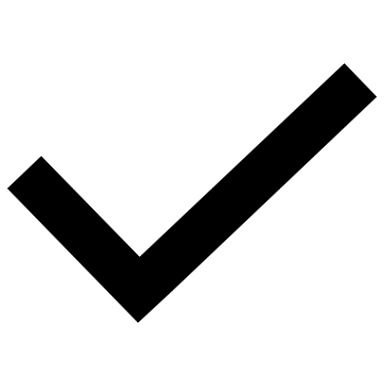 | 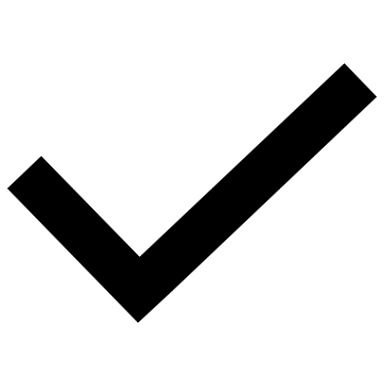 | – | – | – | 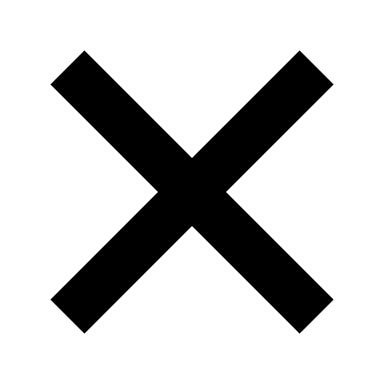 | 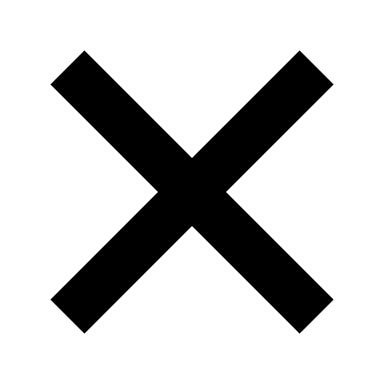 | 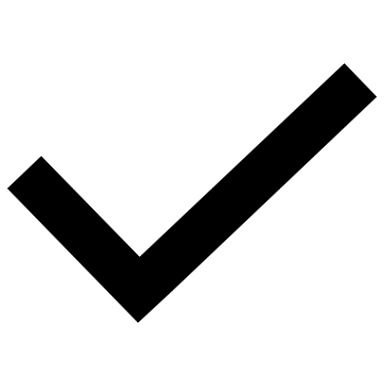 | 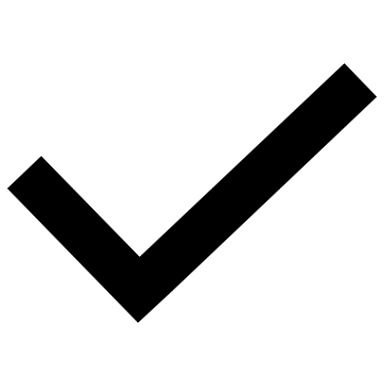 | 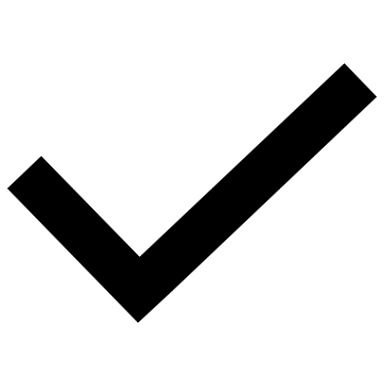 |
| Hinton et al (2023) | 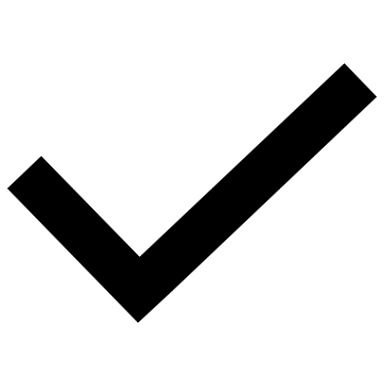 | 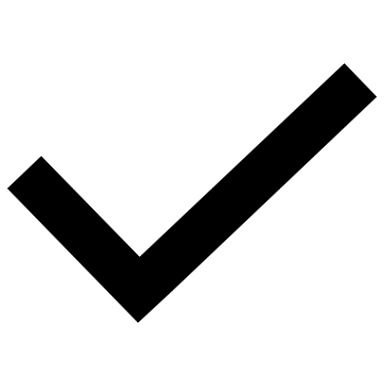 | 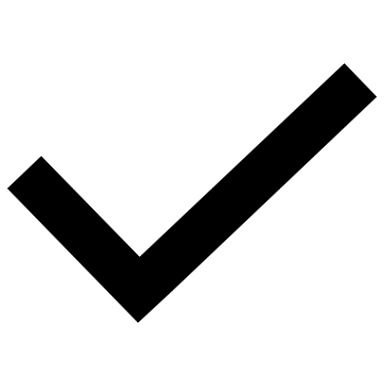 | 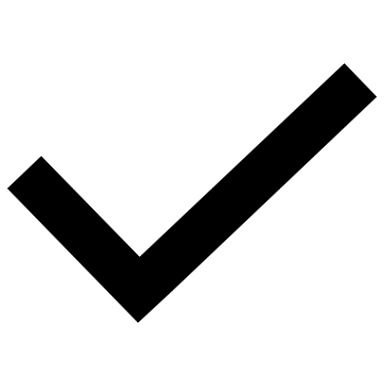 | – | 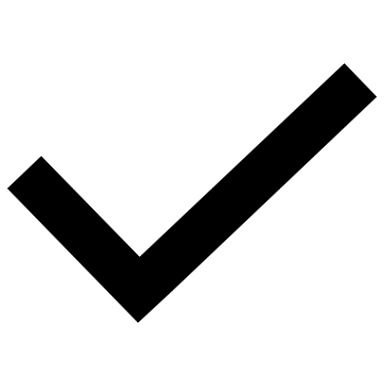 | 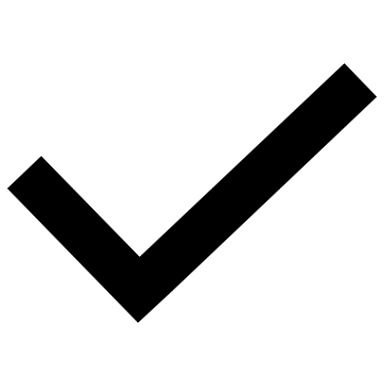 | – | – | 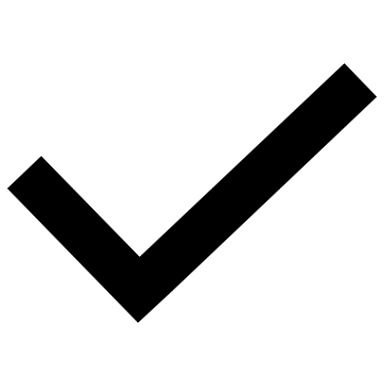 | 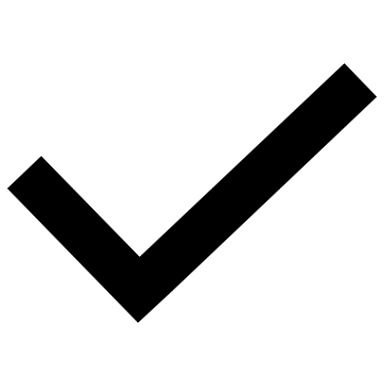 | – |
| Jones et al (2022) | 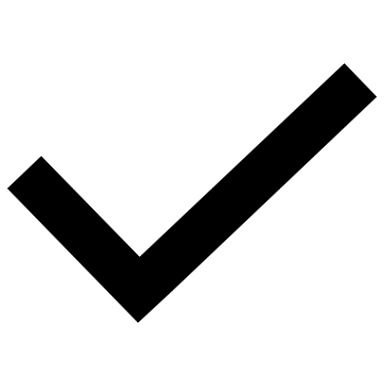 | 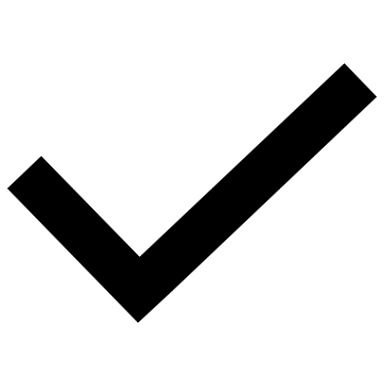 | 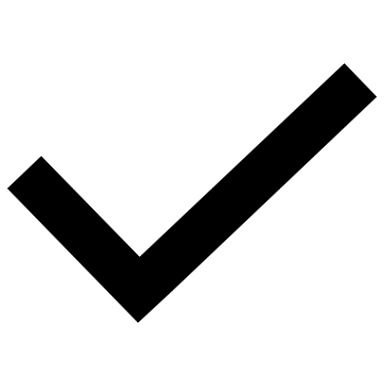 | – | 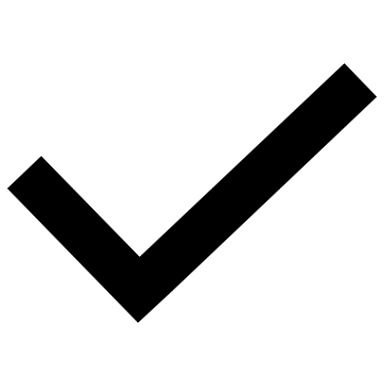 | 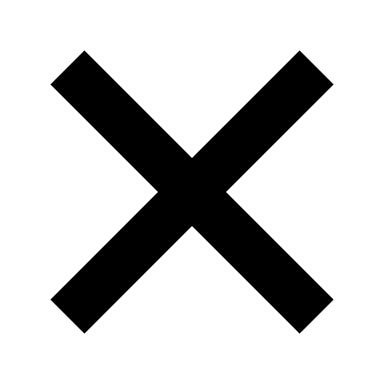 | 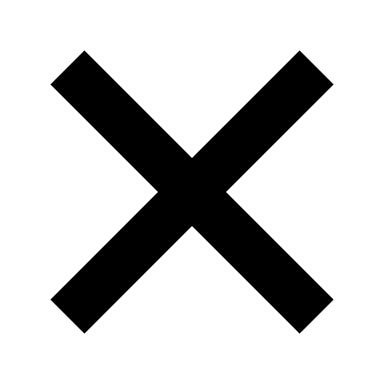 | 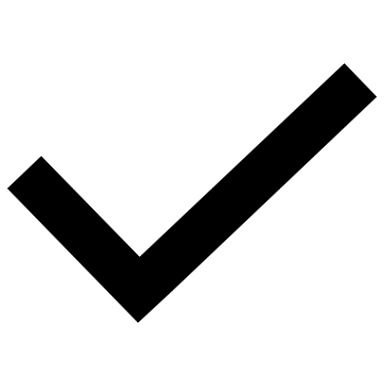 | 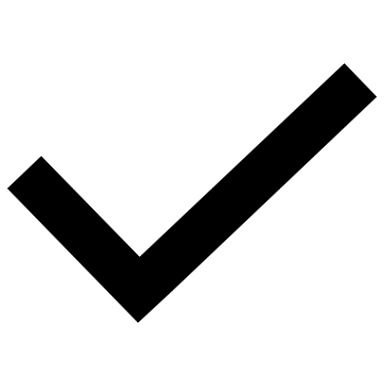 | 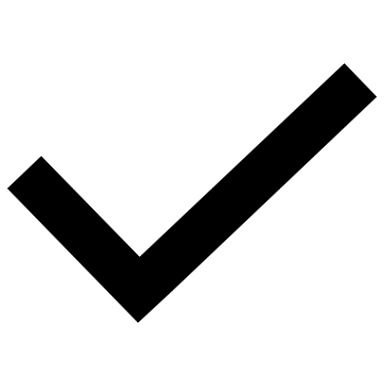 | 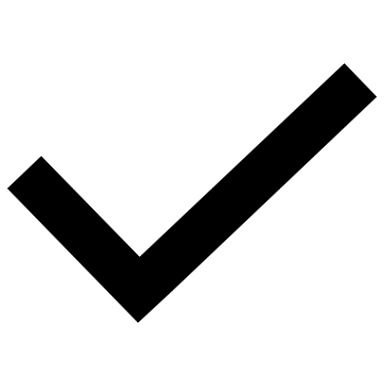 | 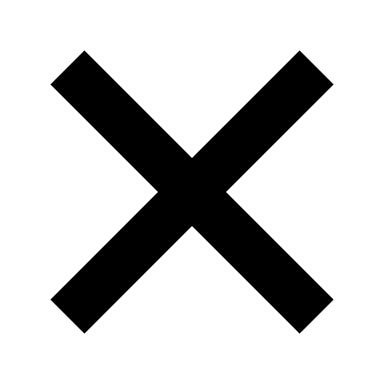 |
| Martin-Key et al (2021) | 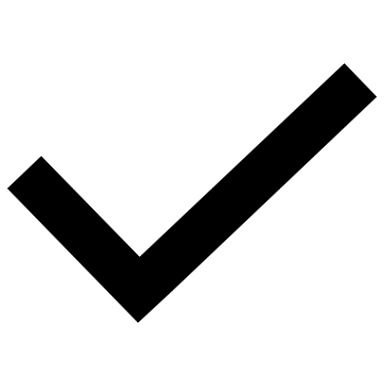 | 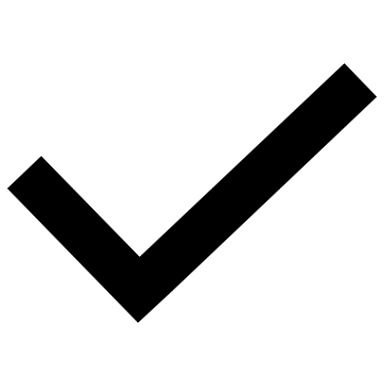 | – | – | 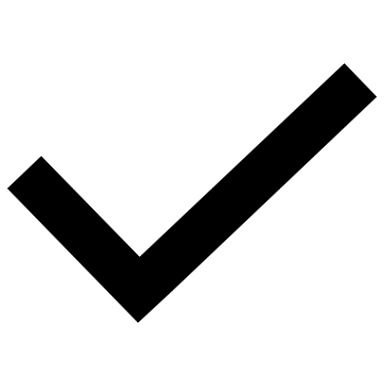 | 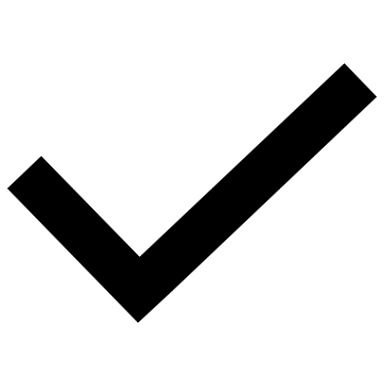 | 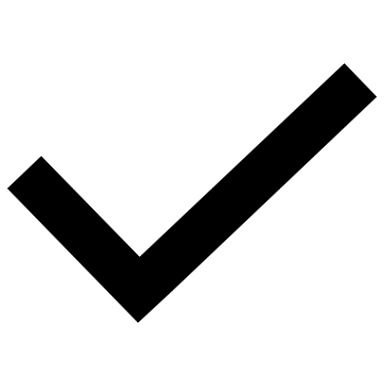 | 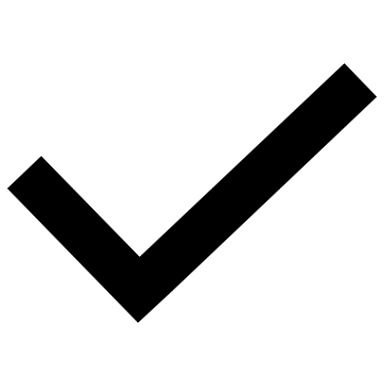 | 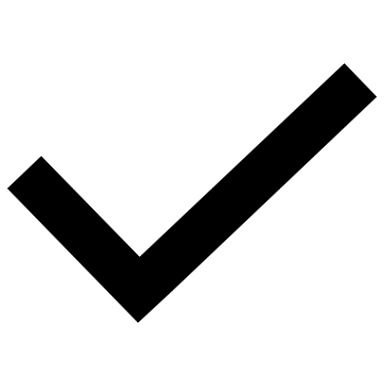 | 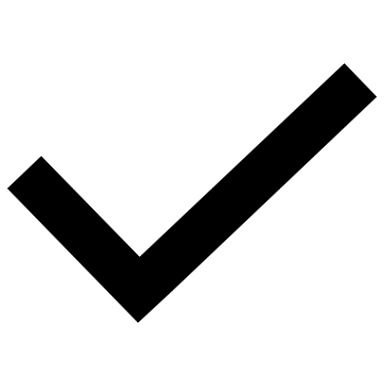 | 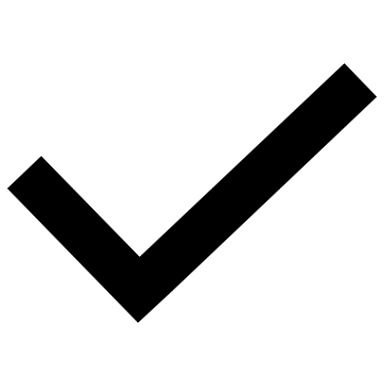 | 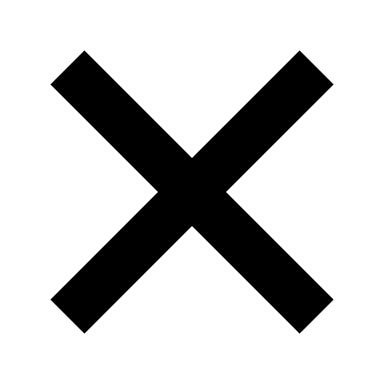 |
| Moltrecht et al (2022) | 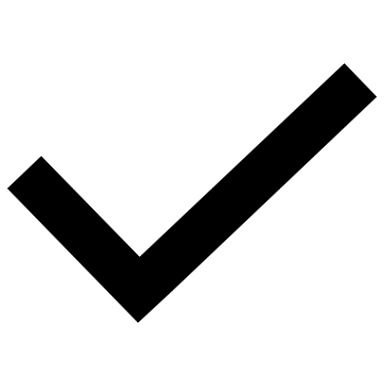 | 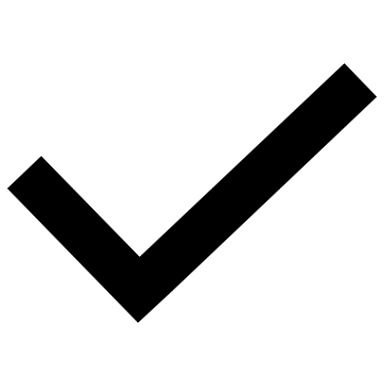 | 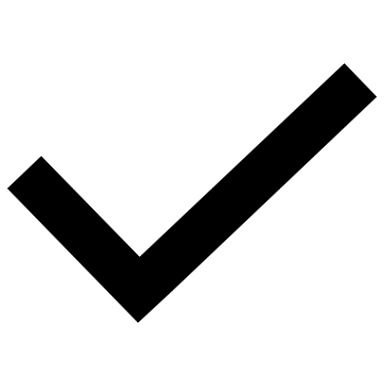 | – | 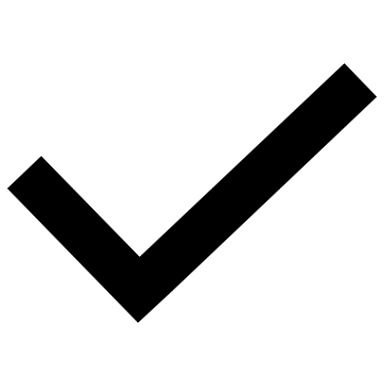 | 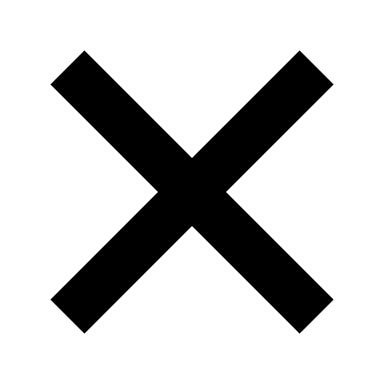 | 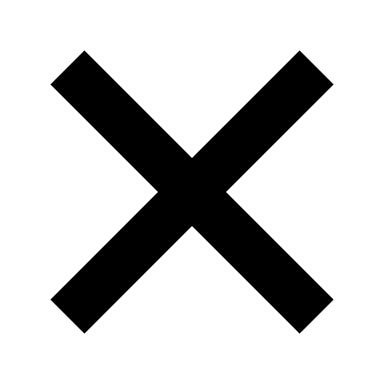 | 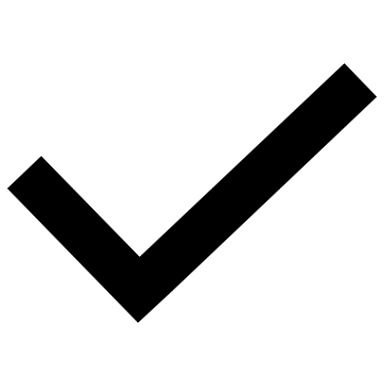 | 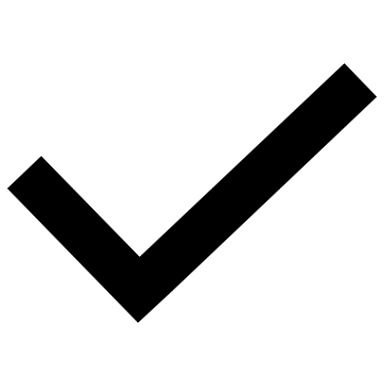 | – | 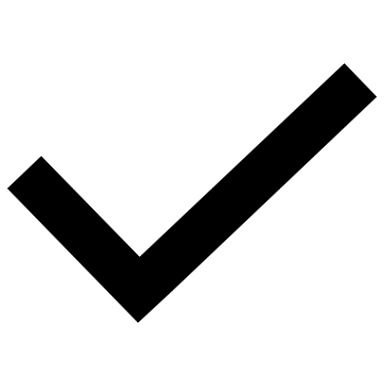 | 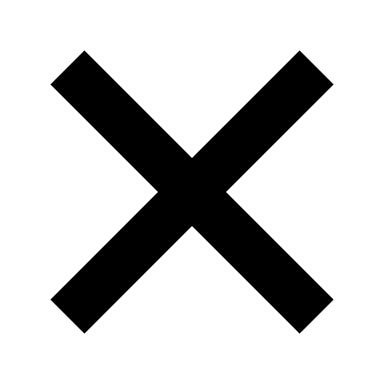 |
| Silverio et al (2022) | 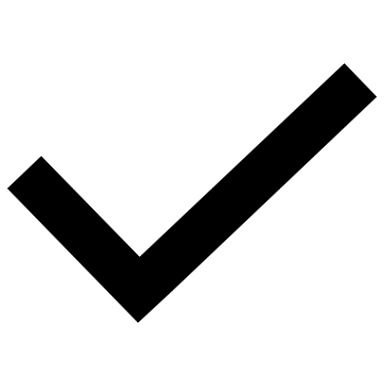 | 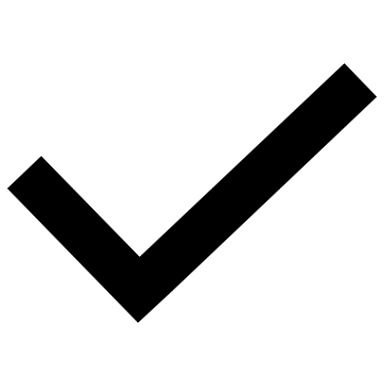 | 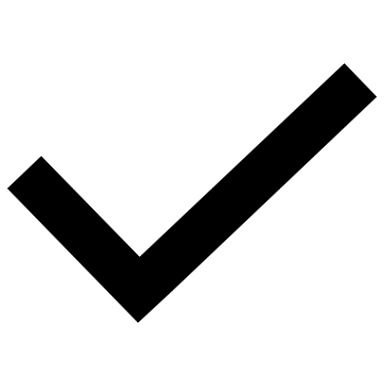 | 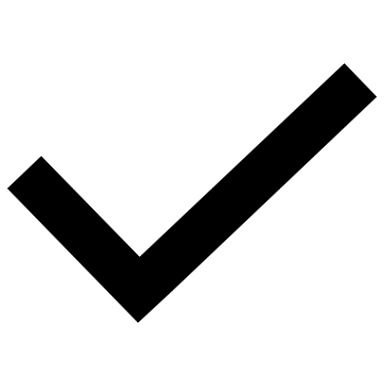 | 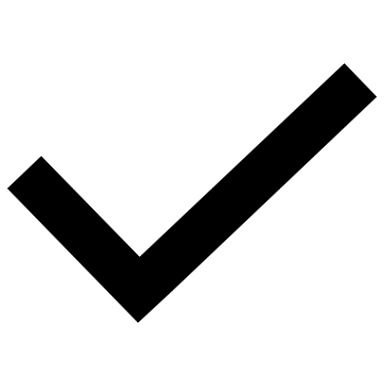 | 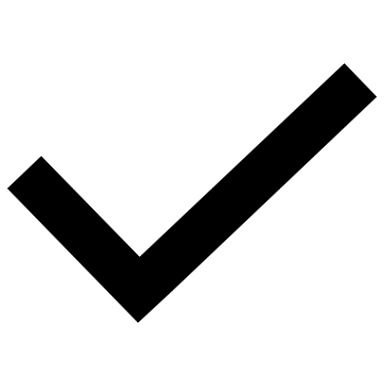 | 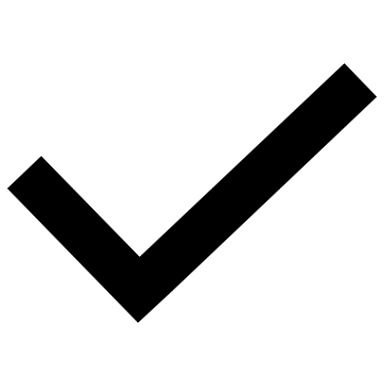 | 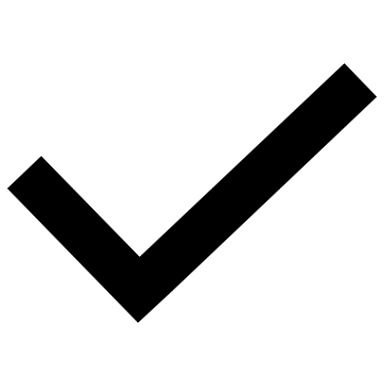 | 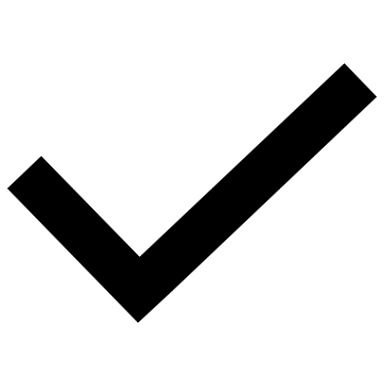 | 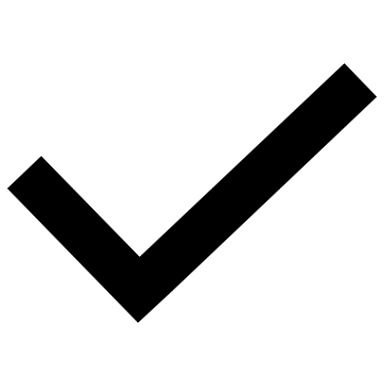 | 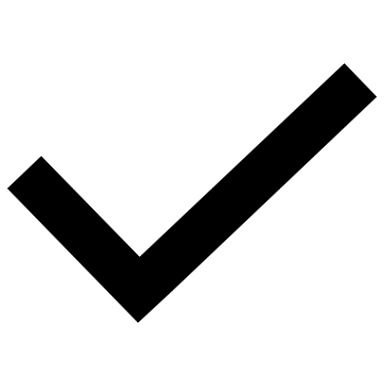 | 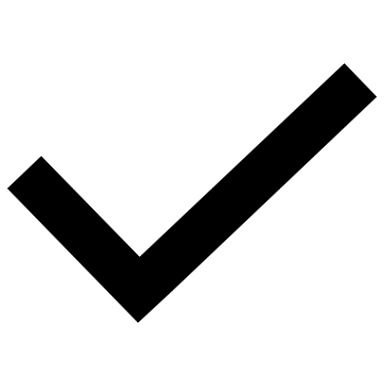 |
| Silverio et al., (2023) | 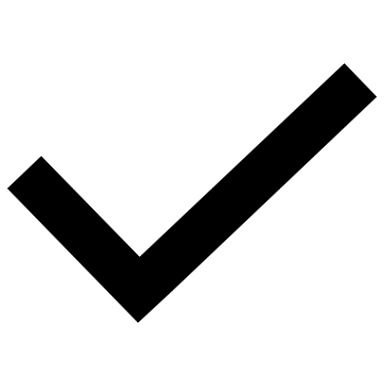 | 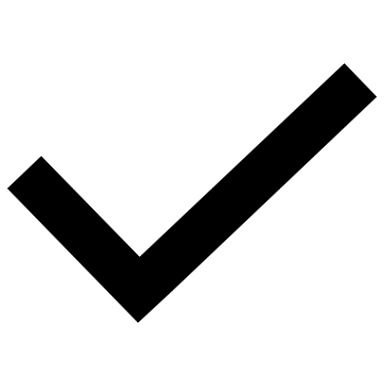 |  |  |  |  |  |  |  |  |  |  |
| Thomson et al (2022) |  |  |  | – |  | – | – |  |  |  |  |  |
| van den Berg et al (2022) |  |  | – |  |  | – |  | – |  |  |  | – |
| Wilson et al (2021) |  |  |  | – |  |  |  |  |  |  |  |  |
| Wiseman et al (2022) |  |  | – |  | – |  |  | – | – |  |  |  |

*A ‘tick’ indicates that a criterion was fully-met, ‘–’ that the criterion was partially-met, and an X that the criterion was not met
