# Supplementary material for: Healthcare providers’ experiences of maternity care service delivery during the COVID-19 pandemic in the United Kingdom: a follow-up systematic review and qualitative evidence synthesis
Source: Front Glob Womens Health. 2024 Nov 28;5:1470674. doi: 10.3389/fgwh.2024.1470674 (PMC11634857; doi:10.3389/fgwh.2024.1470674)
Supplement: Supplementary file 5 [file Table5.docx]

**Table S5:** Process of theme development according to RESILIENT concepts

| **Table S5: Process of theme development according to RESILIENT concepts** | | |
| --- | --- | --- |
| **RESILIENT concept** | **Theme (references)** | **Descriptive code** |
| Care-seeking and care experience | Changes to existing care^a^  (10, 11, 12, 15, 17, 18, 19, 20, 21, 23, 24) | Reduction in midwifery-led care  Closure of community-based perinatal services  Reduced choice in birth plan  Changes in visiting regulation  Lack of person-centred care  Changes as a result of staffing shortages  Increasing use of technology to promote engagement with care |
|  | Limitations placed on the partner^c^  (11, 13, 15, 16, 19, 20) | Inconsistency in protocols  Absence throughout women’s care  Negative birth experiences for women  Unaddressed parental mental health |
|  | Mental health and lack of support networks^b^  (10, 12, 15, 16, 17, 18, 19, 21, 24) | Reduced staff morale  Increased staff workload  Increased need for perinatal mental health support  Concerns in how to support women’s mental health  Staff neglection of own mental health  Care-providers self-sacrificing |
|  | Barriers to implementation of reconfiguration strategies^b^  (11, 12, 15, 17, 18, 20, 22, 23, 24) | Preexisting staff shortages and overstretched services  Increased work demands  Finite resources  Lack of guidance  Time constraints and discontinuity of care as a barrier to mental health monitoring  Lack of ethnic equity |
| Virtual care | Impact on quality of care^b^  (11, 13, 14, 16, 17, 23, 24) | Quality of relationships  Assessment of mental health or domestic violence  Child safeguarding |
|  | Increased convenience and flexibility^c^  (14, 16, 17, 21, 22, 23, 24) | Perceived for women:  Reduced need for childcare,  Reduced travel time  Reduced waiting times  Privacy to discuss sensitive issues  Increased frequency of contacts  For care-provider:  Patients easier to react  Reliance on dedication of individual care-providers  Often limited time due to job planning |
|  | Digital exclusion^b^  (13, 14, 16, 17, 23) | Access to internet  Access to electronic devices  Language barriers  Digital skills |
| Ethical future of maternity care | Optimising patient care^n^  (12, 13, 14, 16, 17, 21, 23, 24) | Hybrid blend of virtual and in-person care  Increased autonomy for women  Address unequal access to technology and increasing access |
|  | Patients and staff as the driving force for change^n^  (10, 11, 12, 13, 14, 15, 16, 18, 19, 20, 21, 23, 24) | Co-design approach with patients and staff  Provisions to encourage open discussions  Involvement of risk groups  Overall improvement in quality of care  Realistic changes in line with staff experience |
| a Mapped theme to Flaherty *et al.* (2022) theme: Altered maternity care  b Mapped theme to Flaherty et al. (2022) theme: Personal and professional impact  c Included in themes derived from Women’s experiences, but not HCP data in original review (Flaherty *et al.* (2022))  n Not included in results of the original review ((Flaherty *et al.* (2022) | | |
